# Complications after transcatheter aortic valve implantation using transfemoral and transapical approach in general anaesthesia

**DOI:** 10.1371/journal.pone.0193558

**Published:** 2018-04-13

**Authors:** Fabian Würschinger, Sigrid Wittmann, Sophia Goldfuß, Nina Zech, Kurt Debl, Michael Hilker, Bernhard M. Graf, York A. Zausig

**Affiliations:** 1 Klinik für Anästhesiologie, Universitätsklinikum Regensburg, Regensburg, Deutschland; 2 Medizinische Klinik II, Intensivmedizin, Universitätsklinikum Regensburg, Regensburg, Deutschland; 3 Klinik für Herz-Thorax-Chirurgie, Universitätsklinikum Regensburg, Regensburg, Deutschland; University of Bern, University Hospital Bern, SWITZERLAND

## Abstract

**Background:**

Transcatheter aortic valve implantation (TAVI) is a minimally invasive procedure used to treat degenerative heart valve disease. The implantation requires a highly specific and interdisciplinary management approach. Currently, TAVI is performed with the patient under local or general anaesthesia.

**Methods:**

This study was a retrospective analysis of all TAVI procedures performed at the University Hospital of Regensburg between January 2009 and July 2015. All pre-, intra and postoperative data focusing on perioperative complications were recorded.

**Results:**

A total of 853 transfemoral- and transapical-TAVI patients were included in the study. All patients underwent general anaesthesia. The ASA classifications were primarily 3–4. The average logistic EuroScores for the transfemoral- and transapical-TAVI patients were 18 ± 12% and 21 ± 15% (p = 0.002), respectively. The anaesthesia coverage time was 170 ± 49 min., including 37 ± 12 minutes for anaesthetic management. Overall, 458 complications were recorded; with pneumonia, acute renal failure, indication for a permanent pacemaker and non-extubation in the operating theatre the most frequently recorded complications.

**Conclusion:**

In the present study, we showed that our patients’ outcomes are comparable to those reported in the available literature. Compared to TF, TA patients show an overall worse physical condition as well as a higher perioperative morbidity and mortality. Consequently TA patients need additional care and should only be operated in appropriately experienced medical centres.

## Introduction

The most common form of heart valve disease in western countries is aortic valve stenosis. [1] Its prevalence among patients older than 65 years of age is 2% to 7%. [2] Etiologically, the most common cause is valve calcification. [1] Surgical aortic valve replacement is currently the gold standard for treating aortic valve stenosis in low- or intermediate-risk patients. [3]

Transcatheter aortic valve implantation (TAVI) is a minimally invasive surgical procedure that was initially developed as an alternative treatment for patients with a high level of perioperative risk, and it has since established itself in this field. [4] TAVI involves the implantation of a prosthetic valve mounted on a stent and introduced with a catheter through transfemoral (TF), transapical (TA), transaxillary/subclavian or direct transaortic access. [5] Usually, the TF approach is preferred, because thoracotomy and penetration of the myocardium are not needed. The TA approach is common, if severe atherosclerotic disease does not allow retrograde insertion of the catheter. [6] The first TAVI was performed by Cribier et al. in 2002. [7] In 2010, 4,859 TAVIs were performed in Germany. In 2013, minimally invasive procedures (10,441) exceeded open surgical (9,899) procedures for the first time. [8] Because the number of surgical interventions remained virtually constant during this time, TAVI was used for patients for whom only a conservative approach had been previously available. While the first TAVI was performed under monitored anaesthetic care (MAC), general anaesthesia (GA) was typically utilized in the early years. [7] In recent years, TAVIs, through TF access, have often been performed under local anaesthesia and MAC in Germany. [8] For anaesthesiologists, TAVI is challenging for many reasons. First, many patients have a serious pre-existing condition and these are therefore high-risk patients. [5] Second, these patients are exposed to marked hemodynamic fluctuations during the TAVI procedure, for example, due to functional heart arrest through rapid pacing. To mitigate the risk of cardiopulmonary decompensation, a highly specific and interdisciplinary management approach is required, possibly including the deployment of extracorporeal membrane oxygenation (ECMO). [9]

For the present article, all TAVI interventions performed at our hospital using TF and TA access were evaluated retrospectively. The goal was to collect key information about the different types of operations, typical complications and procedural parameters in the context of benchmarking and to compare these results with the current literature.

## Materials and methods

### General information, data collection and definitions

After obtaining the consent of the University of Regensburg’s Ethics Commission, all TA- and TF-TAVI patients who had been treated at the University Hospital Regensburg during the period of interest, namely, January 2009 to July 2015, were retrospectively included. TAVIs using other types of access were excluded. The consulted sources included anaesthesia records (Medlinq®, Hamburg, Germany), data from the patient data management system (PDMS, Metavision®, Tel Aviv, Israel) and intensive care unit (ICU), as well as records, medical reports and quality management (QM) data from the hospital information system (SAP®, Walldorf, Germany). The period of observation ended with the discharge of the patient from the hospital. The data were collected in a standardized and anonymized format.

#### Preoperative data

Preoperative data from the anaesthesia records and medical reports included demographic data, such as age, sex, height, weight, American Society of Anesthesiologists (ASA) class, logistic EuroScore (European System for Cardiac Operative Risk Evaluation), New York Heart Association (NYHA) class, long-term medication, haemoglobin level and left-ventricular ejection fraction (EF). The patients’ previous medical history was examined for conditions such as chronic kidney disease (CKD), cerebrovascular events (CVEs), including stroke and transient ischemic attacks (TIAs), myocardial infarction (MI), chronic obstructive pulmonary disease (COPD), diabetes mellitus (DM), and pre-existing cardiac arrhythmias.

#### Intraoperative data

Key elements of intraoperative data included the selected access type, date of the operation, prosthesis type, anaesthetic procedure, cardiopulmonary resuscitation (CPR), use of ECMO, transfusion of erythrocyte concentrates and extubation rate, as well as the induction, procedure, and emergence durations. The study periods were defined as follow: “induction” from the start of the anaesthetic treatment until entering of the hybrid operating theatre, “procedure time” from the skin incision to the closure of the incision, and “emergence” from the closure of the incision to the end of the anaesthetic treatment. The “anaesthesia coverage time” was defined as the time from the start to the end of the anaesthetic treatment. Surgical complication data were also collected, including pericardial effusion, annular rupture, ventricular perforation, vascular complication of the access vessels, bleeding, valve dislocation, and balloon rupture. CVEs, MIs and procedure changes were also documented.

#### Postoperative data

Postoperative data included the patient’s stay in the ICU (ICU records, QM data), IMC (intermediate care, IMC records, QM data), and stay in the general ward (medical reports, QM data). Such data included the ventilation duration, respective length of stay, transfusion of erythrocyte concentrates, and implantation of a permanent pacemaker (PM), as well as complications, such CVE, MI, acute kidney injury (AKI), pneumonia and sepsis. The mobilization time describes the period from admission to the ICU to the first mobilization out of bed. The mortality data reference in-hospital mortalities. Mortality reasons are divided into cardiac, AKI, CVE, bleeding and infections. Cardiac reasons include MI, cardiac arrest, ventricular fibrillation and heart failure. Bleeding includes retroperitoneal bleeding, ventricular perforation, haematothorax and gastrointestinal bleeding.

### Description of the procedure

All patients were admitted and evaluated at least one day before the operation. TAVIs were performed by the cardiac team (cardiac surgeon, cardiologist, and cardiac anaesthetist) in a hybrid operating theatre. All licensed valve types were deployed. Anaesthesia was induced with etomidate (Etomidat-Lipuro®, B. Braun Melsungen AG, Melsungen, Germany), remifentanil (Ultiva®, GlaxoSmithKline GmbH & Co. KG, Munich, Germany) and rocuronium (Rocuronium Inresa®, Inresa Arzneimittel GmbH, Freiburg, Germany) and maintained with sevoflurane (Sevorane®, AbbVie Deutschland GmbH & Co. KG, Wiesbaden, Germany). Norepinephrine (Arterenol®, Sanofi-Aventis-Deutschland GmbH, Frankfurt a. Main, Germany) was continuously administered through intravenous infusion at the discretion of the attending to achieve an adequate circulatory support. A prophylactic antibiotic (1.5 g Cefuroxim Hikma®, Hikma Pharma GmbH, Gräfelfing, Germany) was administered to each patient. In the operating theatre, the patient was connected to a defibrillator, and a TEE probe was introduced. After preparing the access points and anticoagulation with heparin (Ratiopharm GmbH, Ulm, Germany; mean given dose: 5293 ± 2643 IU), the native valve was opened under rapid ventricular pacing (RVP), and the prosthesis was implanted. The position and function of the prosthesis was verified with TEE. Extubation of the patient was the goal at the end of each procedure. After surgery, patients were monitored for at least 12 hours in the ICU or IMC. Following this period, patient care continued either in the ICU or in the general ward.

### Data processing

The data were analysed with SPSS (IBM Corp., Armonk, USA, version 23) by applying Pearson’s chi-squared test, t-tests, Mann-Whitney-U-Test and logistic regression. A p-value of < 0.05 was considered to be statistically significant. All data in the text, tables and figures are specified as a percent (%) or otherwise described in detail.

## Results

During the time period investigated, 853 TF- and TA-TAVI patients were included in the study. Data for 100% of these patients were analysed. TF access was selected for 506 patients (59%), with TA chosen for 347 patients (41%). The demographic data and pre-existing conditions are shown in Table 1. The TA patients’ average logistic EuroScore (TA: 21 ± 15% vs. TF: 18 ± 12%; p = 0.002) and their ASA-class (p = 0.033) were significantly higher than of the TF patients’. The elective use of ECMO was similarly frequent in both groups (TF: n = 24 (5%), TA: n = 15 (4%); p = 0.868).

**Table 1:** Demographic data.

	all	TF: n = 506 [59]	TA: n = 347 [41]	p
Age (years)	79 ± 6	79 ± 6	79 ± 6	0.238
Gender (female)	448 [53]	278 [55]	170 [49]	0.094
BMI (kg/m^2^)	27 ± 5	27 ± 5	27 ± 5	0.496
ASA Classification	3	3	3	0.033*
1	0	0	0	
2	7 [1]	3 [1]	4 [1]	
3	574 [67]	357 [71]	217 [63]	
4	264 [31]	141 [28]	123 [36]	
5	3 [0]	2 [0]	1 [0]	
Not specified	5 [1]	3 [1]	2 [1]	
NYHA Classification	3	3	3	0.394
1	32 [4]	21 [4]	11 [3]	
2	211 [25]	134 [27]	77 [22]	
3	404 [47]	244 [48]	160 [46]	
4	103 [12]	62 [12]	41 [12]	
Not specified	103 [12]	45 [9]	58 [17]	
Logistic EuroScore (%)	19 ± 14	18 ± 12	21 ± 15	0.002*
EF (%)	54 ± 13	54 ± 13	53 ± 13	0.185
Normal (≥55%)	507 [59]	311 [62]	196 [57]	
Slightly reduced (45–54%)	142 [17]	86 [17]	56 [16]	
Moderately reduced (30–44%)	121 [14]	67 [13]	54 [16]	
Severely reduced (<30%)	54 [6]	29 [6]	25 [7]	
Not specified	29 [3]	13 [3]	16 [5]	
History of CVE	138 [16]	81 [16]	57 [16]	0.925
History of myocardial infarction	65 [8]	34 [7]	31 [9]	0.239
Pacemaker pre-existing	105 [12]	72 [14]	33 [10]	0.044*
DM	305 [36]	173 [34]	132 [38]	0.275
COPD	114 [13]	65 [13]	49 [14]	0.609
CRF	309 [36]	175 [35]	134 [39]	0.246

TF: transfemoral; TA: transapical; BMI: Body Mass Index; ASA: American Society of Anesthesiologists; NYHA: New York Heart Association; EF: Ejection fraction; CVE: cerebrovascular events; DM: Diabetes mellitus; CRF: chronic renal failure

All data are presented as the mean ± standard deviation, median or number [%].

*p < 0.05.

Overall, TA patients had significantly more complications per patient than TF patients (0.7 vs. 0.4; p = 0.001). Subsequently, the complications are addressed in detail. All complications are summarized in Table 2.

**Table 2:** Complications.

	Total	TF (n = 506)	TA (n = 347)	p
All (n)	458	226	232	
Mean per patient	0.5	0.4	0.7	0.001*
Conversion rate to SAVR	9 [1]	4 [1]	5 [1]	0.498
Vascular complications	35 [4]	28 [6]	7[2]	0.013*
CVE	20 [2]	9 [2]	11 [3]	0.249
MI	7 [1]	5 [1]	2 [1]	0.707
AKI	89 [10]	27 [5]	62 [18]	<0.001*
Pneumonia	62 [7]	29 [6]	33 [10]	0.044*
Sepsis	17 [3]	5 [1]	12 [4]	0.022*
Permanent PM	106 [12]	59 [12]	47 [14]	0.46
In-hospital Mortality	50 [6]	19 [4]	31 [9]	0.003*
ECMO emergency	22 [3]	16 [3]	6 [2]	0.272
CPR	41 [5]	25 [5]	16 [5]	0.872

CVE: cerebrovascular events; MI: Myocardial infarction; AKI: acute kidney injury; PM: permanent pacemaker. All data are presented as number [%].

*p < 0.05.

### Vascular complications

Vascular complications (e.g., bleeding, hematoma, dissection) arising from the intervention were observed in 35 patients (4%). Such complications were significantly more frequent for the TF patients than for the TA patients (6% vs. 2%; p = 0.013).

### CVEs

A postoperative CVE occurred in 20 patients (2%). There was no significant difference in the frequency of CVEs between the TF and TA patients (2% vs. 3%; p = 0.187).

### MIs

Seven patients (1%), five TF patients and two TA patients experienced an MI during the study period. Two TF patients and one TA patient had an MI during surgery. CPR was applied on two occasions–once before RVP and once during RVP. In the third patient, ST-segment elevation occurred after release of the prosthetic valve, without any serious haemodynamic impairment. The vessel occlusions were each confirmed by coronary angiography. The remaining four MIs occurred postoperatively. Three of these MIs expressed themselves as an increase in cardiac enzymes on the same day as surgery or the day after, with one MI being preceded by severe bleeding. In the case of the fourth patient, haemodynamic instability occurred after a few days, with echocardiography revealing a dislocation of the prosthesis leading to coronary occlusion. The incidence of MIs in patients with surgical complications was significantly higher than in patients without surgical complications (3.7% vs. 0.5%; p = 0.022).

### AKIs

An AKI occurred in a total of 89 patients (10%). The occurrence was significantly higher in the TA patients than in the TF patients (TA: n = 62 (18%) vs. TF: n = 27 (5%); p < 0.001). Patients who suffered an AKI had a significantly higher average EuroScore (27% vs. 18%; p < 0.001) and a significantly lower average initial haemoglobin level (11.7 g/dl vs. 12.4 g/dl; p = 0.004). In addition, they received 1.2 ± 2.9 erythrocyte concentrates on average during their hospital stay, whereas patients without AKI received 0.4 ± 1.6 erythrocyte concentrates on average (p = 0.007). Overall, 100 (12%) patients received erythrocyte concentrates.

### Pneumonia

A total of 62 patients (7%) developed postoperative pneumonia. Patients with pneumonia had a significantly longer stay at ICU (122 h vs. 38 h; p < 0.001) and in hospital (19 d vs. 10 d; p <0.001). With each day that the patients spent in the hospital, the risk of contracting pneumonia increased by 6% (p < 0.001). With each hour that the patients spent in the ICU, the risk of contracting pneumonia increased by 1% (p < 0.001). TA patients had a significantly higher rate of pneumonia than TF patients (10% vs. 6%; p = 0.044). Patients who could not be extubated in the operating theatre had a significantly higher rate of pneumonia than those who were extubated immediately (15% vs. 6%; p = 0.004) and patients who had a ventilation > 48 h had a significantly higher risk of contracting pneumonia than those who had a ventilation < 48 h (46% vs. 7%; p = 0.001). Non-extubation was mostly due to interventional complications or side-effects. Overall, 10% of TF patients and 14% of TA patients could not be extubated in the operating theatre (p = 0.083).

### Permanent pacemaker

A total of 59 TF patients (12%) and 47 TA patients (14%; combined total 106 = 12%) required postoperative fitting of a permanent PM. 72 TF Patients (14%) and 33 TA Patients (10%) already had a PM before TAVI (p = 0.044). The need for postoperative implantation of a PM was significantly higher in the patients with first-degree atrioventricular block (AV block), incomplete left bundle branch block (LBBB) or right bundle branch block (RBBB). 21% of patients with first-degree AV block and 12% of patients without first-degree AV block needed a permanent PM (p = 0.029). 37% of patients with incomplete LBBB and 12% of patients without incomplete LBBB needed a PM (p < 0.001). 28% of patients with RBBB and 12% of patients without RBBB needed a PM (p = 0.001). Six patients had preoperative second-degree AV block. Three of these patients had already had a PM fitted, and a further patient required a permanent PM after the TAVI. The need for a permanent PM was also significantly higher following implantation of a Medtronic CoreValve (CoreValve Revalving System, Medtronic, Minneapolis, USA) (n = 28 (21%) vs. n = 63 (10%); p = 0.001). Apart from Medtronic CoreValve (n = 134 (16%)), we used Edwards SAPIEN (Edwards Lifesciences Corporation, Irvine, USA) (n = 420 (49%)) and Symetis Valves (Boston Scientific Corporation, Marlborough, USA) (n = 219 (26%)). In the rest of the cases (n = 80), the valve type could not be gathered from our sources.

### Conversion rate

Four TF patients (0.8%) and five TA patients (1.4%) were switched to an open surgical procedure. The reasons for open surgery in the four TF patients were an embolization of the prosthesis into the ascending aorta, a perforation of the ventricle, and implantation problems due to calcification of the aortic root. The fourth case could not be determined retrospectively. The reasons for open surgery in TA patients were severe regurgitation of the prosthesis in two cases, a highly fragile myocardium in one patient, and a dislocation of the prosthesis in two cases.

### Mortality

**Fig 1:**
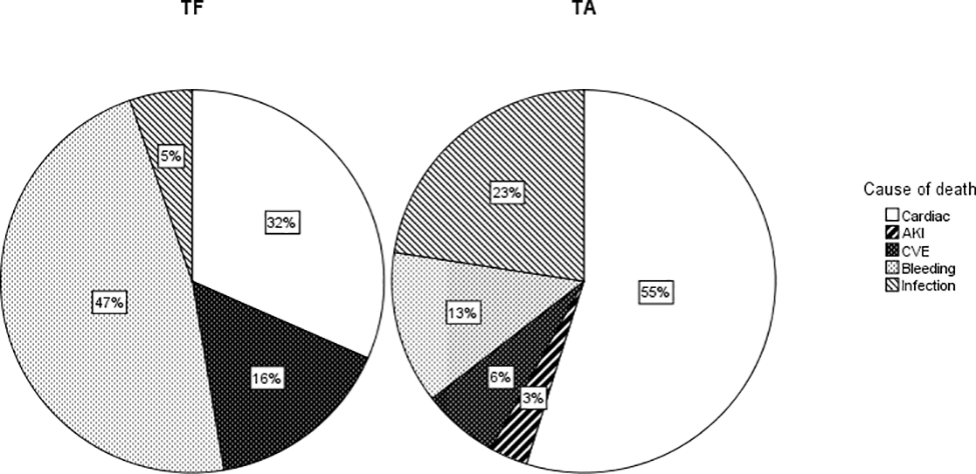
Causes of death (in-hospital) with TF (transfemoral) and TA (transapical) access. All data are presented as percentage. P-values were calculated from raw data: Cardiac (MI, acute heart failure and cardiac arrest): p = 0.148; Acute kidney injury (AKI): p = 1; cerebrovascular events (CVE): p = 0.355; bleeding: p = 0.018; infection: p = 0.134.

The overall hospital mortality rate in our study was 6% (50 patients). There were 19 deaths among the TF patients and 31 deaths among the TA patients (n = 19 (4%) vs. n = 31 (9%); p = 0.002). The causes of death divided into TF and TA are shown in Fig 1. TF patients died significantly more often by bleedings (n = 9 (47%) vs. n = 4 (13%); p = 0.018). CVEs were also a more frequent death cause in TF patients, but without reaching statistical significance (n = 3 (16%) vs. n = 2 (7%); p = 0.355). TA patients died more often due to cardiac reasons (including MI, acute heart failure and cardiac arrest) or infections than TF patients, but also without reaching statistical significance.

### Time specifications

The average anaesthesia coverage time for TA and TF was 174 ± 49 min. The mean anaesthesia coverage time for TA access (180 ± 50 min) was significantly longer than that for TF access (170 ± 48 min; p = 0.007). However, no difference was observed between TA and TF in terms of the induction time (average: 37 ± 12 min), the procedure time (average: 87 ± 44 min) or the emergence time (average: 7 ± 7 min). Significant differences between TF and TA were observed concerning the length of stay at ICU (TF: 35 ± 52 h, TA: 57 ± 84 h; p < 0.001), IMC (TF: 17 ± 51 h, TA: 60 ± 92 h; p < 0.001) and in hospital (TF: 10 ± 7 d, TA: 14 ± 11 d, p < 0.001) and the time to mobilization (TF: 20 ± 18 h, TA: 34 ± 37 h; p < 0.001) (Table 3).

**Table 3:** Time specifications.

	all	TF (n = 506)	TA (n = 347)	p
Anaesthesia coverage time (min)	174 ± 49	170 ± 48	180 ± 50	0.007*
Induction time (min)	37 ± 12	37 ± 12	39 ± 12	0.131
Procedure time (min)	87 ± 44	86 ± 45	91 ± 43	0.106
Emergence time (min)	7 ± 7	7 ± 7	8 ± 8	0.353
Length of ICU stay (h)	24 (21–38)	24 (21–27)	24 (21–65)	<0.001*
Length of IMC stay (h)	0 (0–46)	0 (0–0)	18 (0–98)	<0.001*
Length of hospital stay (d)	9 (7–14)	8 (7–13)	10 (7–15)	<0.001*
Time to mobilization (h)	19 (11–29)	16 (8–24)	23 (18–42)	<0.001*

All data are presented as the mean ± standard deviation or as median (IQR).

*p < 0.05.

## Discussion

This retrospective study enrolled data of 853 TAVI patients. TA access was associated with a higher ASA-status, EuroScore and complication rate. AKI, pneumonia sepsis and mortality were more often documented in TA group than TF group. In contrast, vascular complications occurred more frequently in the TF group. Length of ICU, IMC and total hospital stay was prolonged in the TA group.

The preoperative assessment revealed a higher ASA status and EuroScore for TA patients than for TF patients. A higher ASA status corresponds to a higher incidence of perioperative morbidity and mortality. [10] The EuroScore is the most widely used risk index for open cardiac surgery, and it may be seen as an indicator for the TA patients to have a higher probability to die due to the surgery. Although, this tool is primarily validated for open heart surgery, at this moment it is also accepted to evaluate the TAVI risk. [11] Taken together, the anaesthesiologic, as well as the operative risk for TA patients was higher than for TF patients. The increased length of stay on ICU, on IMC or in hospital may be attributed to this increased assessed risk by itself. However, in the TA group more complications were documented, which might even influence the length of the hospital stay. It has to be kept in mind, that the TF approach is commonly the first choice when a TAVI is under consideration. Only, if the condition of the patient’s peripheral vessels does not allow TF TAVI, the TA approach is chosen, leading maybe to an overall sicker patient population. [12]

In the TF group, vascular complications were 6%. In contrast, TA patients had a significantly lower rate of vascular complications (2%; p = 0,013). These numbers correspond to those described in others studies. There, vascular complications occurred in 4–16.4% of TF-TAVI procedures and 1–4% for TA-TAVI procedures [13–16]. Commonly, in TF access ilio-femoral vessels are damaged mainly due to the delivery system (6). However, except for vascular complications, in the TA group more complications were documented.

Most prominent was AKI in the TA group (TA: 18%, TF: 5%; p < 0.001). Whereas these number correspond to numbers presented in other studies. There, the incidence of AKI is up to 21.9% in TF patients and up to 44.4% in TA patients. [15,17] Barbash et al. have investigated the predictors for AKI, specifically for TAVI. [18] The sole independent risk factor identified by Barbash et al. was the transfusion of erythrocyte concentrates. In our study, the patients who developed an AKI had a preoperative haemoglobin level that was significantly lower than the level in patients who did not develop an AKI (11.7 g/dl vs. 12.4 g/dl, p = 0.004). In addition, the intraoperative and postoperative transfusions of erythrocyte concentrates were associated with a significantly increased incidence of AKI. Previous research has already shown that the transfusion of erythrocyte concentrates does not constitute an adequate treatment of perioperative anaemia in all cases. Indeed, the transfusion is associated with increased morbidity and mortality rates. Early diagnosis and prevention of anaemia is recommended in the context of patient blood management programs to ensure a more targeted and effective deployment of transfusions. [19] Overall, the AKI patients in our study were a subgroup featuring a particularly poor preoperative state of health (average EuroScore: 27% vs. 18%, p < 0.001).

In terms of pneumonia and TAVI, we identified only a single study from Covello et al., which reported a pneumonia rate of 7–8%. [20] Our incidence of pneumonia was also 7% (TF 6% vs. TA 10%; p = 0.044). According to Lynch et al., the risk factors for hospital-acquired pneumonia include mechanical ventilation for > 48 h and a long length of stay in the ICU and the hospital. [21] Our study confirmed these risk factors. According to Hausman et al., COPD patients are generally exposed to a higher risk of pneumonia for non-cardiosurgical interventions. [22] In our study, the COPD patients also tended to contract pneumonia more frequently (11% vs. 7%, p = 0.172), although the incidence was not statistically significant.

According to the literature, the rate of CVE in TF- and TA-TAVI patients is 1–3.8%. [13–17] Differences in the incidence of CVEs between TF and TA have not been described. In our study, 2% of TF patients and 3% of TA patients suffered a CVE, which is comparable to the literature data.

The incidence of MIs in TAVI patients is up to 1.5%. [13,14,17] In our study, the incidence was 1%, with no difference between TF (1%) and TA (1%). Although, due to the design of the study a distinction of the type of MI was not possible, there was a statistically significant association between the complications due to intervention and MI. The incidence of MIs in patients with complications caused by intervention was 3.7%, while only 0.5% of patients without interventional complications suffered an MI (p = 0.022).

According to the literature, 8.7–28.7% of TF and TA patients need a permanent PM. [13–15] Differences between TF and TA are not reported, which is supported by our findings (TF: 12%, TA: 14%; p = 0.46). Studies have cited existing conduction disorders as a risk factor for postoperative PM dependency for TAVI and for conventional aortic valve replacement. [23,24] In our study, this finding was supported by the association between an existing first-degree AV block, an RBBB and an incomplete LBBB and the implantation of a permanent PM. Typically, patients with a higher-degree AV block at the preoperative stage had already been fitted with a PM. It is assumed that the TAVI procedure may cause mechanical damage to the conduction system due to the anatomical proximity to the aortic valve of a prosthesis that is implanted too deeply. [25] Also, the valve model by itself has an influence on the PM incidence.

According to the literature, a procedure change from a TAVI to an open surgical intervention occurs in 0–1% of cases. [13,14] In our study procedure changes were necessary in 1% of cases and mainly resulting from surgical complications.

The mortality rate of TF patients ranges from 4.2% to 8.7%. [13,14,17] The mortality rate of our TF patients (4%) is comparatively low. The TA patient mortality rate was significantly higher (9%). In the literature, similarly high mortality rates for TA patients (8–8.8%) can be found. [15,16] In accordance with our results, Panchal et al. reported in their meta-analysis a higher mortality in TA. [26] As in our study, they found a higher logistic EuroScore in the TA group, which might have been linked to higher all-cause mortality. However, other possible explanations, e.g. a more invasive nature of this approach and its steeper learning curve might be attributable for increased mortality in TA approach.

A review of the literature for TF access revealed procedure times of 75 ± 40 min to 167.6 ± 5.2 min. [17] The major discrepancies between the studies can probably be attributed to the lack of a uniform definition of procedure time. In our study, the average procedure time was 87 ± 44 min (TF: 86 ± 45 min, TA: 91 ± 43 min). According to the literature, the postoperative stay for TF-TAVI patients ranges from 3 ± 4.45 d to 15.5 ± 10.34 d. [13,14,17] For our TF patients, the average length of hospital stay was 10 d. The TA patients spent a significantly longer time in the hospital (14 d) than the TF patients. Our average length of hospital stay appears to be relatively long, but it has to be considered, that the length of stay is strongly dependent on the local health care system, e.g. the DRG system.

## Conclusions

In the present study, we showed that our patients’ outcomes are comparable to those reported in the available literature and that TA patients have an overall worse physical condition as well as a higher perioperative morbidity and mortality. Consequently TA patients need additional care and should only be operated in appropriately experienced medical centres.

## Limitations

During the study period, the proportion of TF-patients in the collective continuously grew to 74.4% in 2015. Furthermore, operative techniques developed over the years and led to a significant reduction of complication rates and significant better patient outcome. Therefore, comparative interpretation of data between the two access groups must be cautious. Another limitation is that we did not use the standardized endpoint definitions for transcatheter aortic valve implantation (VARC-2). [27] urthermore, as we extracted the diagnosis of AKI, MI, CVE or pneumonia directly from our patient data management system, our retrospective study design did not allow us to discriminate between the different types. Additionally, only those data are presented that were documented in the files that were evaluated. Therefore, a comparison with other studies might be limited.
